# Effect of Investment in Malaria Control on Child Mortality in Sub-Saharan Africa in 2002–2008

**DOI:** 10.1371/journal.pone.0021309

**Published:** 2011-06-30

**Authors:** Yoko Akachi, Rifat Atun

**Affiliations:** 1 Strategy, Performance and Evaluation Cluster, The Global Fund to Fight AIDS, Tuberculosis and Malaria, Geneva, Switzerland; 2 Imperial College Business School, Imperial College, London, United Kingdom; Kenya Medical Research Institute - Wellcome Trust Research Programme, Kenya

## Abstract

**Background:**

Around 8.8 million children under-five die each year, mostly due to infectious diseases, including malaria that accounts for 16% of deaths in Africa, but the impact of international financing of malaria control on under-five mortality in sub-Saharan Africa has not been examined.

**Methods and Findings:**

We combined multiple data sources and used panel data regression analysis to study the relationship among investment, service delivery/intervention coverage, and impact on child health by observing changes in 34 sub-Saharan African countries over 2002–2008. We used Lives Saved Tool to estimate the number of lives saved from coverage increase of insecticide-treated nets (ITNs)/indoor residual spraying (IRS). As an indicator of outcome, we also used under-five mortality rate. Global Fund investments comprised more than 70% of the Official Development Assistance (ODA) for malaria control in 34 countries. Each $1 million ODA for malaria enabled distribution of 50,478 ITNs [95%CI: 37,774–63,182] in the disbursement year. 1,000 additional ITNs distributed saved 0.625 lives [95%CI: 0.369–0.881]. Cumulatively Global Fund investments that increased ITN/IRS coverage in 2002–2008 prevented an estimated 240,000 deaths. Countries with higher malaria burden received less ODA disbursement per person-at-risk compared to lower-burden countries ($3.90 vs. $7.05). Increased ITN/IRS coverage in high-burden countries led to 3,575 lives saved per 1 million children, as compared with 914 lives in lower-burden countries. Impact of ITN/IRS coverage on under-five mortality was significant among major child health interventions such as immunisation showing that 10% increase in households with ITN/IRS would reduce 1.5 [95%CI: 0.3–2.8] child deaths per 1000 live births.

**Conclusions:**

Along with other key child survival interventions, increased ITNs/IRS coverage has significantly contributed to child mortality reduction since 2002. ITN/IRS scale-up can be more efficiently prioritized to countries where malaria is a major cause of child deaths to save greater number of lives with available resources.

## Introduction

An estimated 8.8 million children under-five die each year [1]. Countdown to 2015 for Maternal, Newborn, and Child Survival monitors coverage of priority interventions aimed at achieving the Millennium Development Goals (MDGs) 4 (Reduce child mortality) and 5 (Reduce maternal mortality). Of the 68 Countdown priority countries that account for over 90% of maternal and child deaths worldwide, 19 countries are on track to meet MDG 4 while other countries have either experienced insufficient or decelerating progress [2]. Infectious diseases, especially pneumonia, diarrhoea, and malaria are the major causes of death in children aged under-five. In 2008, 16% of under-five deaths in Africa were due to malaria and 4% to AIDS, as compared with global figures 8% and 2% respectively [3].

Investment in HIV, tuberculosis and malaria to achieve MDG 6 (Combat HIV/AIDS, malaria, and other diseases) increased substantially since the creation of The Global Fund to Fight AIDS, Tuberculosis, and Malaria (Global Fund) in 2002 [4], which by the end of 2010 had approved $21.7 billion and disbursed $13 billion. These investments have funded high impact interventions such as prevention of mother-to-child transmission (PMTCT) of HIV, antiretroviral therapy (ART) for children, along with insecticide-treated nets (ITNs) and indoor residual spraying (IRS) to prevent malaria. However, the impact of these investments and expanded ITN/IRS coverage on child health in sub-Saharan Africa has not been quantified.

In this paper, we examined increases in ITN distribution and ITN/IRS coverage following international investments to strengthen malaria control and reduce under-five mortality. Our study focused on 34 sub-Saharan African countries. Africa accounts for 68% of global incidence and 69% of global prevalence of AIDS, and 85% of global malaria incidence [5]. Around 4.2 million of the 8.8 million child deaths in 2008 were in Africa [1]. That year, 92% (0.677 million) of under-five child deaths from malaria were in Africa with five sub-Saharan African countries, Nigeria, Democratic Republic of Congo, Uganda, Sudan, and Tanzania, accounting for 57% (0.417 million) of these deaths [3].

## Methods

We combined data from multiple sources (Table 1) to examine the relationship between financing for malaria control, malaria prevention interventions targeting children, and their impact on child health. As input measure, we used Official Development Assistance (ODA) disbursement for malaria control. For output, we used number of ITNs distributed each year and ITN/IRS coverage. For outcome, we estimated number of lives saved from malaria-attributed deaths among children under-five in addition to under-five mortality rate.

**Table 1:** Data Sources.

Data	Source
Official Development Assistance (ODA) Gross Disbursement for “12262: Malaria control” total and Global Fund, in 2008 constant US$	Credit Reporting Systems (CRS) database; OECD's Development Assistance Committee (DAC)^1^
Number of ITNs distributed	World Malaria Report 2009
Cause of child deaths 2000 & 2008	Child Health Epidemiology Reference Group (CHERG)^2^
Population/under-five of age population	Population Division of the United Nations^3^
Under-five child mortality rate	United Nations Inter-agency Group on Mortality Estimation (IGME)^4^
Under-five child mortality rate	Institute for Health Metrics and Evaluation (IHME)^5^
Essential care during delivery/birth coverage (% of women with essential care during delivery and immediate newborn care)	LiST Data on facility based birth and skilled birth attendance from DHS/MICS and MPS WHR-05, if otherwise unavailable
Stunting (% stunted 0–1 month)	LiST Calculated from WHO or DHS
Access to quality water coverage (% of homes with improved water)	LiSTChildinfo.org or DHS/MICS
DTP3 vaccine coverage (proportion of infants having received 3 doses of diphtheria, tetanus, and pertussis vaccine prior to the survey)	LiST UNICEF MICS
Measles vaccine coverage (proportion of infants having received 2 dose of measles containing vaccine (MCV) prior to the survey)	LiST UNICEF MICS
Hib vaccine coverage (proportion of infants having received 3 doses of Haemophilis influenza type B vaccine prior to the survey	LiST UNICEF MICS
ORS coverage (% of children with diarrhea given ORS from sachets)	LiST DHS
No PMTCT interventions	AIMUNAIDS/WHO/UNICEF AIM module in Spectrum^6^
ART for children coverage	AIMUNAIDS/WHO/UNICEF AIM module in Spectrum
ITN/IRS coverage (% of households with at least 1 ITN or covered by IRS)	LiST MICS/DHS/MIS

Intervention coverage data for the regression analysis was extracted from LiST model.^7^LiST uses baseline data mainly from Demographic and Health Surveys (DHS),^8^Malaria Indicators Survey (MIS),^9^Multiple Indicator Cluster Surveys (MICS)^10^as shown in the table above.

1 http://stats.oecd.org/index.aspx?r=842905 (Accessed August 5, 2010).

2 http://cherg.org/projects/underlying_causes.html (Accessed August 5, 2010).

3 http://www.un.org/esa/population/ (Accessed August 5, 2010).

4 http://www.childinfo.org/mortality_igme.html (Accessed August 5, 2010).

5 http://www.healthmetricsandevaluation.org/resources/datasets/2010/mortality/results/child/child.html (Accessed August 5, ^,^2010).

6 http://www.jhsph.edu/dept/ih/IIP/list/manuals/AIMManual.pdf (Accessed August 5, 2010).

7 http://www.jhsph.edu/dept/ih/IIP/list/manuals.html for details intervention on coverage data sources (Accessed August 5, 2010).

8 http://www.measuredhs.com/start.cfm (Accessed August 5, 2010).

9 http://www.measuredhs.com/aboutsurveys/mis/start.cfm (Accessed August 5, 2010).

10 http://www.unicef.org/ceecis/resources_10594.html (Accessed August 5, 2010).

### Input

We used the Credit Reporting Systems (CRS) database of the Organization for Economic Cooperation and Development (OECD) Development Assistance Committee (DAC) (Box S1), which provides gross commitments and disbursements of ODA, to measure financing provided [6]. In contrast to earlier studies, we used disbursement rather than commitment data because of the wide discrepancy between the two (Box S2). We utilized ODA gross disbursements data from 2002 to 2008, period corresponding with Global Fund investments, when the coverage ratio on CRS disbursements, which measures the comprehensiveness of aid activity data, reached 90% in 2002 (60% before this year) and nearly 100% from 2007 [6].

To analyze ODA trends for 2002 to 2008, we adjusted disbursement data based on 2008 constant US$ (last updated in April 2010) to account for inflation and exchange rate variations. We computed total disbursements and Global Fund share of contributions to malaria control using CRS purpose code “12262: Malaria control”. DAC CRS data includes financing from bilateral and multilateral donors (Box S1), but not the financing from domestic, private or philanthropic sources.

### Output

We analysed malaria interventions and specifically the distribution and coverage of ITNs, the most prominent malaria preventive measure for large-scale deployment in highly endemic areas [7], and IRS used in malaria vector control. Anti-malarial treatment was not analysed in this paper due to lack of data, changes in recommended treatment over time, definition of “correct” treatment, limited scale up in the period studied of artemisinin based combination treatment funded by donors, and country variations in these issues that limit comparison across countries and over time.

We used World Health Organization (WHO) and Roll Back Malaria estimates [8] for the number of ITNs (including long-lasting insecticidal nets, LLINs) distributed each year in countries by ministries of health and other agencies.

We obtained intervention coverage data from Lives Saved Tool (LiST) [9] (Box S3), which uses nationally representative household surveys, including Demographic and Health Surveys (DHS), Malaria Indicators Survey (MIS), and Multiple Indicator Cluster Surveys (MICS), to derive coverage levels. Our objective was to estimate the impact of ITN/IRS coverage on child mortality, controlling for other key health interventions such as immunization affecting health of children under-five. Vaccines and Vitamin A supplementation coverage was obtained using WHO/UNICEF consensus data [9]. For measles coverage, the percentage protected was calculated using the WHO Department of Immunization, Vaccines, and Biologicals model [10]. HIV prevalence, and intervention coverage values were derived from UNAIDS data and updated using UNICEF values [9].

The estimates of neonatal and under-five mortality rates for the LiST base year for each country were obtained using data from United Nations Inter-agency Group for Child Mortality Estimation (IGME) [11]. The indicator of choice for malaria in LiST is household ownership of at least one ITN or covered by IRS [12]. As these malaria control interventions are interlinked, it is difficult to measure their impact separately [13], and data used by LiST combines ITN and IRS. For indicators in which no data were readily available, LiST made linear interpolation between data points [9].

We assumed reductions in under-five child mortality in the period studied were driven by high-impact health interventions [14], [15]. For this analysis, we extracted and used the coverage data for the following interventions from LiST in addition to the stunting data and ITN/IRS coverage: essential care during delivery and birth (% of women with essential care during delivery and immediate newborn care), stunting (% stunted less than one-month old), access to quality water (use of improved water source within 30 minutes), immunizations (DTP3, measles, Haemophilus Influenzae type b (HiB)), access to oral rehydration solution (ORS) (% of children with diarrhoea given ORS from sachets), no PMTCT (% without anti-retroviral prophylaxis to prevent mother-to-child transmission), and ART for children (% of children 0–15 years old receiving ART).

### Outcome

We used two outcome indicators: under-five child mortality rate, and the number of lives saved from malaria-attributed deaths among under-five children from increased coverage of ITN/IRS. We used data from both IGME [11] (also used by LiST) and the Institute for Health Metrics and Evaluation (IHME) [16] to estimate under-five child mortality rate. The correlation between the two measures of under-five mortality rate from IGME and IHME was 0.915. We also used LiST projection to estimate the number of lives saved from malaria-attributed deaths among under-five children from increased coverage of ITN/IRS.

### Lives Saved Tool modelling and projection

To estimate intervention coverage and to analyse the number of lives saved for under-five children from malaria-attributed deaths due to ITN/IRS coverage, we used LiST: a multi-cause model of child mortality incorporated into the Spectrum Policy Models [17], and linked to the Spectrum modules related to demography, HIV/AIDS, and family planning (Box S3). LiST uses country-specific health status information (including nutrition and *Plasmodium falciparum* (*P. falciparum*) exposure), cause of death profile and intervention coverage values, and their changes over time to estimate the changes in cause-specific mortality. The effect sizes used to link intervention coverage and cause-specific mortality were derived using established methods [18], [19]. LiST uses recommended values on intervention efficacy based on international studies and compiles data including the effectiveness of child health interventions in reducing child mortality from diarrhea, pneumonia, measles, and malaria. For example, LiST uses the value 55% as the estimated protective efficacy of ITNs and IRS on reducing malaria-attributable mortality under-five years of age in *P. falciparum* endemic settings [12]. The model enables estimation of cause-specific changes in mortality rates associated with changes in intervention coverage, nutritional status or breastfeeding rates, incorporating changes in population trends due to fluctuations in fertility rates.

### Countries Studied

For the analysis we selected 34 countries in sub-Saharan Africa (See Table 2). We excluded countries in sub-Saharan Africa with small populations (i.e. Cape Verde, Comoros, Sao Tome and Principe, Mauritius, Seychelles), and those lacking at least two nationally representative data points in LiST data sources (Gabon, Equatorial Guinea, Republic of the Congo). Countries that were not part of the Countdown process (Namibia) were also excluded. For malaria and ITN/IRS coverage analysis, we also excluded Botswana, Chad, Lesotho, and South Africa, which received no ODA disbursement for malaria control from 2002 to 2008. We included these countries in the under-five mortality rate analysis as they have high burden of HIV/AIDS and benefit from Global Fund investments for HIV control.

**Table 2:** ODA disbursement, Global Fund investments, ITNs distributed, malaria as a cause of death and lives saved in sub-Saharan African countries in 2002–2008.

Country	ODA Total Disbursement for malaria control (US$ million)	Global Fund disbursement for malaria (US$ million)	# of ITN distributed (million)	Malaria as cause of child death 2000 (%)	Malaria as cause of child death 2008 (%)	Average Under-5 Population (million)	Total # Lives Saved by ITN/IRS coverage change (LiST)
Angola	78.8	20.3	4.9	8.3	8.4	3.2	7943
Benin	26.7	12.1	2.9	27.2	23.3	1.3	4566
Burkina Faso	12.9	8.4	2.3	20.3	20.4	2.7	11678
Burundi	46.8	39.5	3.6	8.4	9.2	1.2	178
Cameroon	29.6	29.5	2.7	22.8	19	2.9	1133
Central African Republic	14.4	13.8	1.5	18.5	14.3	0.7	1751
Congo, Dem. Rep.	82.5	54.3	14	16.9	17	11	10381
Cote d'Ivoire	6.6	4.6	1.6	20.5	21.1	3.1	3431
Eritrea	15.3	12.5	1	13.6	0.3	0.8	1342
Ethiopia	247.1	147.7	25	6.1	6.8	13	3658
Gambia, The	22	21.8	1.3	29.4	23.2	0.3	500
Ghana	66.8	52.3	7.5	33	26.3	3.3	9236
Guinea	10.8	7.1	1	24.5	23.6	1.6	92
Guinea-Bissau	4.5	3.9	0.5	21	17.7	0.2	2999
Kenya	122.7	89.1	23	13.6	10.9	6.2	3061
Liberia	28.3	22.9	1.6		15.6	0.6	2236
Madagascar	80	69.7	7.4	20.1	3.5	2.9	5928
Malawi	51.6	34.4	7.9	14.1	16.6	2.5	10036
Mali	17.1	11.7	4.3	16.9	20.8	2.8	23877
Mauritania	4.3	4.2	0.1	12.2	13.3	0.5	139
Mozambique	69.7	38.8	5.8	18.9	12.5	4.7	27072
Niger	40.7	36	4.4	14.3	18	2.7	29512
Nigeria	104.1	77.9	29	24.1	20.2	25	18450
Rwanda	93.2	71.7	4	4.6	5.9	2.1	809
Senegal	56.4	31.3	3	27.6	18.7	2	9708
Sierra Leone	22.1	12.7	2.6	12.4	12.9	1	11360
Somalia	26.5	25.6	1.3	4.5		1.6	1001
Sudan	91	74.2	6.2	21.2		6	115
Swaziland	1.6	1.6	0.1	0.2	0	0.2	391
Tanzania	199.2	146.4	15	22.7	16.4	6.9	15778
Togo	18.9	18.8	2.5	25.3	25.7	1	3588
Uganda	116.7	91	6.2	23.1	22.4	5.8	6074
Zambia	88.7	68.9	6.2	19.4	15.2	1.9	9730
Zimbabwe	17.7	16.7	1.5	0.2	3.4	1.8	218

To examine the relationship between malaria burden of the country and ODA disbursement made per person-at-risk, we categorized the disease burden into two groups: (i) malaria as cause of child death under-five less than 10% of all cause of deaths (seven of the 34 countries denoted as ‘low-burden’); (ii) 10% or higher (27 of the 34 countries, denoted as ‘high-burden’). Moreover, we divided the 34 countries into quartiles by the cumulative amount of ODA disbursed for malaria control in 2002–2008 divided by at-risk-person for malaria population from year 2007 [20].

For each of the 34 countries analysed, projections of cause-specific changes in mortality rates began from 1990, for which a nationally representative survey had been conducted, except Swaziland where the 1988 survey was used. The cause of death in that year was estimated using methods described by Black and colleagues [3]. Each projection was created to end in 2025, though we use the results for 2002–2008. We chose 2002 as the baseline year and also the first year of intervention program; this is appropriate as the first year is treated as the planning year in LiST and Global Fund was created in 2002 with much of its disbursements made in the subsequent years.

We projected the number of deaths in children under-five years of age between 2002 and 2008 using all available data, including child health intervention coverage, HIV prevalence and stunting rates (observed results). This was compared with an alternative counterfactual scenario which predicts the number of deaths in children under-five between 2002 and 2008 assuming coverage changes in all key health interventions affecting children under-five ***except ITN/IRS coverage*** (ITN/IRS analysis). This projection also included HIV prevalence and stunting changes. Number of lives saved by ITN/IRS coverage was estimated from the difference between the numbers of under-five deaths projected from the ITN/IRS analysis and the numbers of deaths projected from the ‘Observed Results’ for each year. LiST measures the impact of ITN/IRS coverage on reducing malaria-attributable mortality and ***not*** on all-cause child mortality, which would result in a larger number of lives saved.

We examined the relationship between inputs, outputs and the estimated lives saved by plotting the average ITN/IRS coverage rate for 2002–2008 over the number of lives saved per one million children by ITN/IRS scale-up for each of the 34 countries. As indicated, we grouped these countries into two categories according to percentage of child deaths caused by malaria; first, less than 10% (7/34 countries), and the second 10% and higher (27/34 countries). In addition to the average ITN/IRS coverage rate in ***absolute*** terms, we computed the ***change*** in coverage over this period and its impact. By using ordinary least squares (OLS) regression analysis for each country with dependent variable as ITN/IRS coverage and independent variable as year, we estimate the annual trend in ITN/IRS coverage for each country for the years 2002–2008. We then plotted this annual trend in ITN/IRS coverage change over the number of lives saved per one million children by ITN/IRS scale-up for each of the 34 countries.

### Panel data regression analysis

Our data on input, output, and outcome have two elements of information: (i) cross-sectional information reflected in the differences between subjects (34 countries), and (ii) time series information reflected in the changes within subjects over time (2002–2008). The combination of time series with cross-sections enhances the quality and quantity of data in ways not possible using only one of the two dimensions. This enabled us to analyse the relationship between input and output, and the relationship between output and outcome while controlling for between-subject variability, resulting in more efficient estimators of treatment-related effects (e.g. changes in the amount of investment for malaria control on the number of ITNs distributed, or changes in the coverage of ITN/IRS interventions on under-five mortality) when compared to corresponding cross-sectional designs with the same number and pattern of observations [21]. Panel data regression analysis also has the advantage of controlling for omitted variables that differ between countries but are constant over time. By using fixed effects model, we controlled for such omitted variables and for bias that may be country-specific but are constant over time including issues with LiST coverage data and projection results [22], [23]. In our analysis, we used panel data regression techniques to study the effect of input on output and their impact. We used statistical techniques to address limitation and weakness of the available data: for example, fixed effects model to address the omitted variables bias, instrumental variable to address the issues of reverse causality, and feasible generalized least squares with autocorrelation correction to address existence of autocorrelation within panels due to interpolated data.

### Input to output

#### Relationship between ODA disbursements for malaria control on the number of ITNs distributed

In examining the relationship between input and output for malaria control in the 34 countries, we used regression analysis to estimate the number of ITNs associated with US $1 million ODA disbursement for malaria control. As there is a lag between disbursement of funding and implementation, we examined both ODA disbursed one year prior to ITN distribution and ODA disbursed the same year.

We first ran pooled OLS regression analysis ignoring the time series effect. We next ran random effects regression for the years 2002–2008. We also ran fixed effects regressions. For either independent variable (lagged or the same year ODA disbursement), Hausman tests were rejected [22], [23], and fixed effects model was preferred. The null hypothesis was rejected by the F test with either dependent variable (F test that all u_i = 0: F(33, 136) = 3.78 and F(33, 108) = 3.59 respectively, in both cases Prob>F = 0.0000), and we concluded that the fixed effect model is better than the pooled OLS models as there is time-series effect.

### Output to Outcome

#### Impact of ITN distribution on Lives Saved

We examined the relationship between the number of ITNs distributed and the number of lives saved among children under-five-years of age from ITN/IRS coverage change as estimated from LiST.

We ran panel regression with number of under-five lives saved with ITN/IRS scale-up as the dependent variable. We included another variable, malaria as cause of all child deaths in 2008 (%), as proxy for the disease burden of malaria. We first used random effects model and then used fixed effects model. As Hausman test was insignificant, we chose random effects model, a more efficient estimator [22], [23]. Moreover, using the result from the Input to Output analysis described earlier, we used total ODA disbursement for malaria control as an instrumental variable and studied the impact of the number of ITNs distributed on lives saved to address issues of measurement errors and reverse causality in our model. We chose ODA disbursement as instrumental variable because it is correlated with the endogenous explanatory variable (i.e. number of ITNs distributed), and we can assume that it is not correlated with the error term in the explanatory equation because invest ment in malaria control should only affect lives saved among under-five children from malaria-attributed deaths due to ITN/IRS coverage through its output (ITNs distributed).

#### Impact of ITN/IRS coverage on Under-five Mortality Rate

Panel data analysis was conducted for years 2002–2008 for 38 countries to see the impact of major child health intervention coverage change on under-five child mortality rate. We added four countries: Botswana, Chad, Lesotho, and South Africa, which received no ODA disbursement for malaria control during 2002 to 2008 and were thus excluded from the ITN/IRS analysis. We included these countries in the under-five mortality rate analysis as they have high burden of HIV/AIDS and benefit from Global Fund investments for HIV control. We first ran random effects models including other health interventions and stunting data described in the output data section in addition to ITN/IRS coverage to examine how they were associated with under-five mortality. Other health interventions and stunting data were included in the model because we wanted to estimate the impact of ITN/IRS coverage on child mortality controlling for other key factors that influence child mortality. We next used fixed effects model with cluster option. We clustered the standard errors as there is a reason to believe that error terms are not independent in a subgroup (country) of the observations; this is because under-five mortality rates are rarely available annually and are often interpolated between the years. The clustering option, however, ignores the time component, and given the high degree of autocorrelation with annual data, we tested for autocorrelation [24]. We found that the test statistics indicate the presence of serial correlation. Due to lack of reliable data on annual basis, both IGME and IHME interpolated between data points to estimate the under-five mortality rates for the last several decades. Our analysis only studied the past several years, and we can expect that for this short period of time we are focusing on, there is panel-specific first degree autocorrelation [22], [25]. We used feasible generalized least squares to allow for estimation in the presence of autocorrelation within panels. We applied this model to both measures of under-five mortality rates.

## Results

Key data and results for the 34 sub-Saharan African countries are shown in Table 2: with total ODA amount disbursed for malaria control from 2002 to 2008 and the proportion disbursed by the Global Fund in the first two columns; cumulative number of ITNs distributed in the third column; the proportion of all child deaths from malaria for a country in the next two columns for 2000 and 2008 respectively; estimated average population of children under-five-years of age in the sixth column; and the cumulative number of lives saved by expanded ITN/IRS coverage from baseline year 2002 projected by LiST for each country in the final column. This estimate of number of lives saved was the result of LiST projections as explained in detail in the Methods section describing LiST model.

In the 34 countries studied, financing for malaria control as measured by ODA disbursed increased from $9.8million in 2002 (Global Fund contribution $0) to $651.7million in 2008 (Global Fund contribution $347.2million). A total of $1,916 million was disbursed, including $1,372 million of Global Fund investments that comprised over 70% of the total ODA disbursements (from multilateral and bilateral sources) for malaria control.

**Figure 1:**
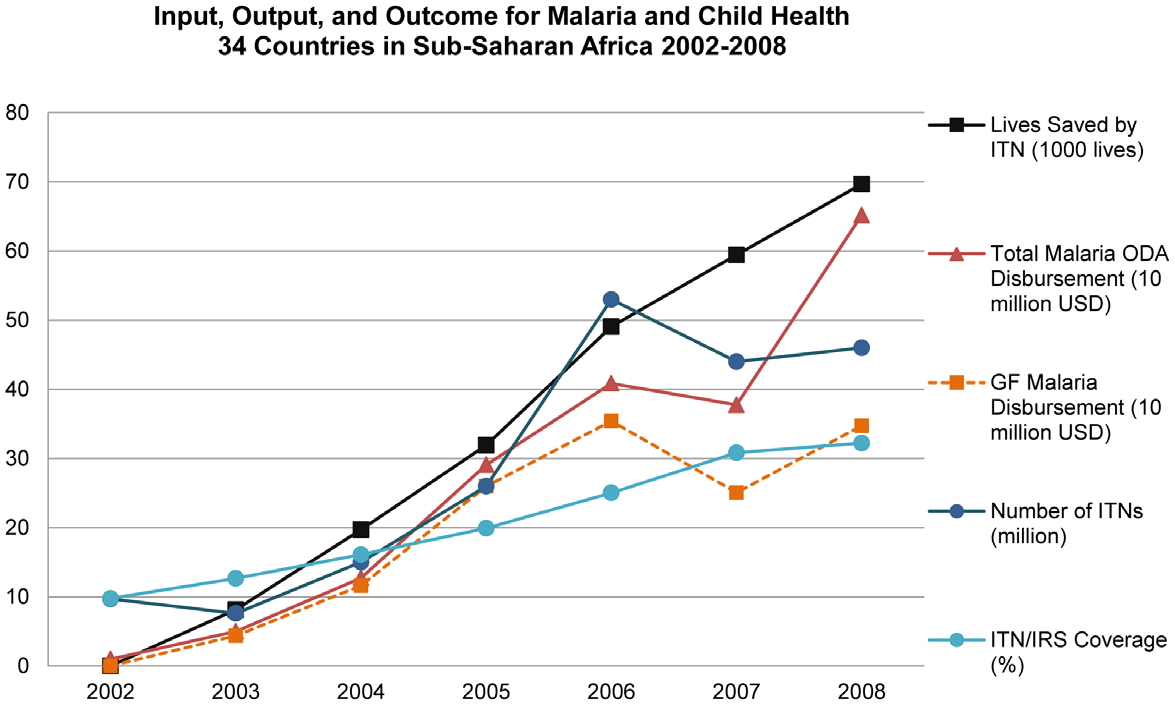
Input, Output, and Outcome for Malaria and Child Health: 34 Countries in Sub-Saharan Africa 2002–2008.

**Figure 2:**
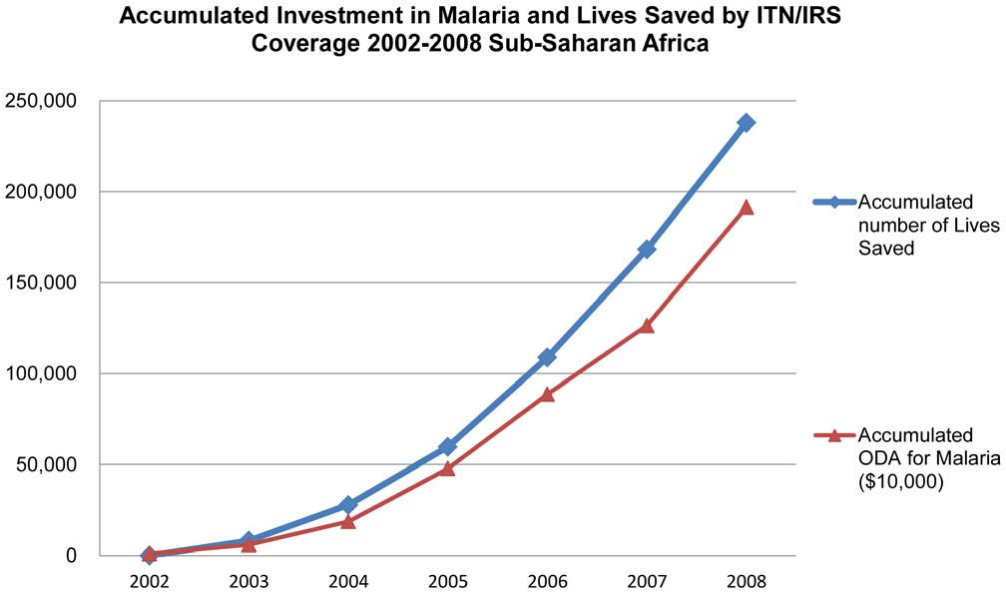
Accumulated Investment in Malaria and Lives Saved by ITN/IRS Coverage 2002–2008 Sub-Saharan Africa.

This financing enabled distribution of 151 million ITNs, increasing from 9.7 million in 2002 to 46 million in 2008. In this period, the average ITN/IRS coverage increased from 8.3% to 33% (Figure 1). Cumulatively, investments and increased coverage with ITN/IRS led to an estimated 240,000 lives saved (Figure 2). Increased ODA disbursement was significantly associated with increased ITN/IRS coverage (p<0.05) when we ran OLS. Use of OLS to determine the association between ODA disbursement for malaria and the number of lives saved due to ITN/IRS coverage increase the same year, showed that ODA disbursement was significant (p<0.0001) indicating that for every $1 million ODA disbursement for malaria control, 31 lives under-five are saved due to ITN/IRS coverage change (Figures 1 and 2).

Analysis of the relationship between ODA disbursement per person-at-risk and the proportion of under-five mortality deaths due to malaria showed (Table 3) that high-burden countries did not necessarily receive higher ODA disbursement per person-at-risk (Burkina Faso, Cameroon, Cote d'Ivoire, Guinea, Nigeria) while some countries where malaria is not a major cause of child deaths received the highest funding per person-at-risk (Angola, Burundi, Ethiopia, Rwanda, Swaziland).

**Table 3:** Malaria burden on children under-five and malaria control ODA disbursement per person-at-risk in 2002–2008.

	Malaria ODA Disbursement 2002–2008 per person-at-risk*			
	Lowest percentile (0–25%)	25–50%	50–75%	Highest percentile (75–100%)
Malaria as cause of all child deaths less than 10% in 2000	Zimbabwe	Somalia		Angola Burundi Ethiopia Rwanda Swaziland
Malaria as cause of all child deaths 10% or higher and less than 20% in 2000	Congo, Dem. Rep Mali	Central African Rep. Mozambique Niger	Eritrea Kenya Malawi Mauritania Sierra Leone	Zambia
Malaria as cause of all child deaths 20% or higher in 2000	Burkina Faso Cameroon Cote d'Ivoire Guinea Nigeria	Benin GhanaGuinea-Bissau Sudan Togo	Madagascar Tanzania Uganda	The Gambia Liberia Senegal

*Cumulative amount of ODA disbursement for malaria control 2002–2008 in the country divided by person-at-risk of malaria 2007 population.

### Input to Output

#### Relationship between ODA disbursements for malaria control on the number of ITNs distributed

For every US$1million ODA disbursed for malaria control each year, about 50,000 ITNs were distributed the same year (Table 4). There is a possibility of a time-lag between ODA disbursement and intervention implementation; when assuming a one-year lag between disbursement and ITN distribution, about 29,000 ITNs were distributed per $1 million ODA disbursement for malaria.

**Table 4:** Relationship between total ODA Disbursement for malaria control and number of ITNs distributed in Sub-Saharan Africa 2002–2008.

	ODA disbursed the same year as ITN distribution	ODA disbursed one year prior to ITN distribution	ODA disbursed the same year as ITN distribution		ODA disbursed one year prior to ITN distribution	
	1	2	3	4	5	6
	OLS	OLS	Random effects	Fixed effects	Random effects	Fixed effects
Number of ITNs per US$1 million ODA for malaria control	68719**(6073)	65778**(8603)	57766**(5928)	50478**(6352)	47685**(8431)	29304**(8851)
N	171	143	171		143	
R-squared	0.4310	0.2931				
within			0.317		0.092	
between			0.569		0.578	
overall			0.431		0.293	
Hausman test			Chi-sq = 10.15; Prob>chi-sq = 0.0014		Chi-sq = 46.53 Prob>chi-sq = 0.0000	

Dependent variable: Number of ITNs distributed in respective year.

Coefficients represent per annum change for the fixed effects and random effects models.

Significance level indicated as *(5%), **(1%).

Based on 34 countries, standard error in parentheses, constant terms abbreviated.

### Output to Outcome

#### Impact of ITN scale up on Lives Saved

The impact of ITN distribution on lives saved and the impact of ITN/IRS coverage on lives saved were analysed separately as these two output indicators differ: the former is the number of ITNs distributed each year in countries by ministries of health and other agencies whereas the latter is the household ownership of at least one ITN or households covered by IRS.

#### Impact of ITN distribution on Lives Saved

Panel regression with number of under-five lives saved (according to LiST projections) with number of ITNs distributed as dependent variable (Table 5), showed that 0.625 lives were saved per 1,000 additional ITNs distributed.

**Table 5:** Relationship between number of ITNs distributed and number of lives saved among under-5 children due to ITN/IRS coverage in Sub-Saharan Africa 2002–2008.

	1	2	3
	Random effects	Fixed effects	Instrumental variable
Lives Saved (estimated from LiST and ITN/IRS coverage) per 1,000 ITN distributed	0.408**(0.063)	0.411**(0.066)	0.625**(0.128)
Malaria as cause of all child death 2008 (%)	47.3(34.7)	(omitted)	45.9(35.3)
N	199	199	161
R-squared			
within	0.191	0.191	0.114
between	0.127	0.070	0.160
overall	0.152	0.119	0.141
Hausman test	Chi-sq(1) = 0.03; Prob>Chi-sq = 0.869		-

Dependent variable: Number of lives saved in children under-five due to ITN/IRS scale-up (estimated from LiST).

Model 3 uses ODA disbursement for malaria as instrument variable for number of ITNs distributed (see Table 2).

#### Impact of ITN/IRS coverage on Lives Saved

**Figure 3:**
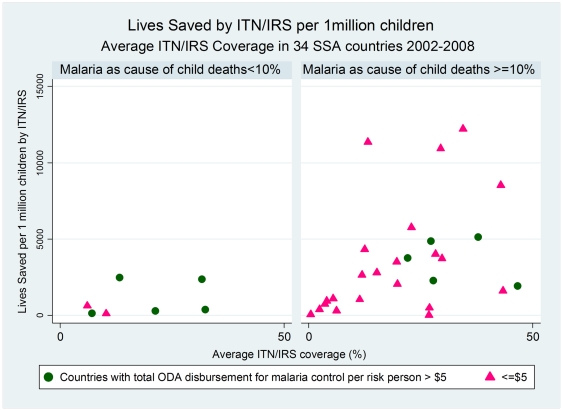
Lives Saved by ITN/IRS per 1 million children: Average ITN/IRS Coverage in 34 SSA countries 2002–2008.

Comparison of average ITN/IRS coverage rate for 2002–2008 with the number of lives saved per 1 million children by ITN/IRS scale-up for each of the 34 countries (Figure 3) shows that more lives were saved in countries with higher-burden of child deaths due to malaria. Countries with lower malaria burden on children (malaria as cause of all child deaths less than 10%) received $7.05 ODA disbursement per person-at-risk, in contrast to countries with malaria as a major cause of child deaths (malaria as cause of all child deaths more than 10%) which received on average $3.90 per person-at-risk (Table 6).

**Table 6:** Comparison between low and high burden countries for malaria on child mortality.

	Mean total ODA disbursement for malaria control 2002–2008 per person at risk	Mean ITN/IRS coverage estimate 2002 (%)	Mean ITN/IRS coverage estimate 2008 (%)	Total lives saved per 1 million under-five children 2002–2008	Total number of lives saved	Total ODA disbursement for malaria control 2002–2008
Low burden: Malaria as cause of all child deaths less than 10% in 2000 (7 countries)	$7.05	5.6	37.0	914	14,198	$512 million
High burden: Malaria as cause of all child deaths 10% or more in 2000 (27 countries)	$3.90	9.1	31.9	3,575	223,773	$1,404 million

7 low burden countries are: Angola, Burundi, Ethiopia, Rwanda, Somalia, Swaziland, Zimbabwe.

**Figure 4:**
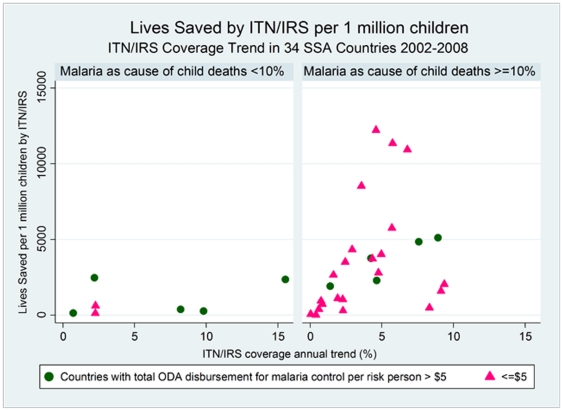
Lives Saved by ITN/IRS per 1 million children: ITN/IRS Coverage Trend in 34 SSA Countries 2002–2008.

In 2002, the ITN/IRS coverage rate in countries with lower burden of child deaths due to malaria was 5.6% and 9.1% in countries with higher burden of child deaths due to malaria. In 2008, the mean coverage rates increased six-fold to 37.0% in lower-burden countries while only tripling to 31.9% in higher-burden countries (Table 6). The average coverage rates for the two groups of countries were similar for the years 2002–2008 (17.4% in low-burden versus 21.2% in high-burden countries), but the change in ITN/IRS coverage rates during this period between the two groups differed; where countries with lower malaria burden on children enjoyed a greater increase in ITN/IRS coverage (from 5.6% to 37.0%) compared to high-burden countries (from 9.1% to 31.9%) (Table 6, Figure 4).

On average, increased ITN/IRS coverage in high-burden countries led to an estimated 3,575 lives saved per 1 million children, as compared to 914 lives in lower-burden countries, despite higher per risk person disbursement of ODA for malaria control for the latter group. In absolute terms, between 2002 and 2008 a total of $512 million were disbursed in the seven low-burden countries leading cumulatively to an estimated 14,198 lives saved compared to $1,404 million disbursed in the 27 high-burden countries leading to 223,773 lives saved (Table 6).

#### Impact of ITN/IRS coverage on Under-five Mortality Rate

For the 38 countries in the period 2002–2008, panel data analysis of coverage changes in major child health interventions and their impact on the under-five mortality rate showed that 10% increase in households with either ITN or IRS would reduce 1.5 child deaths per 1000 live births (column 5, Table 7).

**Table 7:** Relationship between Under-five Mortality Rate and intervention coverage in Sub-Saharan Africa 2002–2008.

	1	2	3	4	5	6
	U5M,IGME	U5M,IHME	U5M,IGME	U5M,IHME	U5M,IGME	U5M,IHME
	Random	Random	Fixed, Cluster	Fixed, Cluster	FGLS, AR(1)	FGLS, AR(1)
Essential care during delivery/birth coverage	0.179(0.166)	−0.229(0.162)	0.215(0.199)	−0.239(0.181)	0.483*(0.227)	0.722**(0.159)
Stunting	0.359(0.234)	−0.047(0.227)	0.229(0.318)	−0.150(0.346)	2.241**(0.308)	1.367**(0.184)
Access to quality water coverage	−0.317**(0.103)	−0.281**(0.099)	−0.240*(0.096)	−0.244(0.202)	−0.606**(0.103)	−0.657**(0.068)
DTP3 coverage	−0.210**(0.055)	−0.295**(0.054)	−0.188*(0.072)	−0.282**(0.044)	−0.711**(0.077)	−0.536**(0.063)
Measles coverage	−0.149**(0.028)	−0.167**(0.027)	−0.151**(0.044)	−0.171**(0.044)	−0.105*(0.044)	−0.046(0.032)
Hib coverage	−0.049**(0.019)	−0.062**(0.018)	−0.052*(0.022)	−0.063(0.262)	−0.003(0.031)	0.003(0.023)
ORS coverage	−0.446**(0.109)	−0.224*(0.105)	−0.441*(0.172)	−0.211(0.262)	−0.043(0.095)	−0.290**(0.076)
No PMTCT interventions	0.148**(0.026)	0.080**(0.026)	0.150**(0.047)	0.081(0.048)	0.098*(0.043)	0.006(0.030)
ART children coverage	−0.104**(0.026)	−0.009(0.026)	−0.103*(0.047)	−0.007(0.026)	−0.075*(0.038)	−0.052(0.028)
**ITN/IRS coverage**	−0.099**(0.033)	−0.108**(0.033)	−0.106*(0.043)	**−0.111(0.061)**	−0.152*(0.062)	−0.174**(0.044)
N	266	266	266	266	266	266
R-squared						
within	0.715	0.646	0.716	0.646		
between	0.442	0.352	0.420	0.338		
overall	0.441	0.363	0.417	0.349		

Dependent variable: Under-five Mortality Rate.

Correlation of the two measures of U5MR is 0.913 for years 2002–2008 for the 38 countries (Botswana, Chad, Lesotho, South Africa are included in the models above in addition to the 34 countries) Significance level indicated as *(5%) **(1%), standard error in parentheses, constant terms abbreviated.

In the same model, improved access to quality water (p<0.01), DTP3 (p<0.01), measles (p<0.05), ART for children had significant (p<0.05) impact on reducing under-five mortality rate, while increase in stunting (p<0.01) and lack of PMTCT (p<0.05) interventions led to significantly worsened under-five mortality rate. Increase in essential care during delivery and birth was negatively correlated with under-five mortality (p<0.05), which is the opposite direction from expected. This was the only variable among all interventions that showed statistical significance in the opposite direction from expected. A 10% increase in access to quality water, DTP3 coverage, measles coverage, and ART for children coverage would lead to a reduction of 6.1, 7.1, 1.1, 0.8 child deaths per 1000 live births respectively. A 10% increase in stunting was associated with a reduction of 22.4 child deaths per 1000 live births. A 10% decline in population PMTCT coverage would lead to a reduction of 1.0 child death per 1000 live births.

DTP3 and measles vaccines were significant in all models. The impact of ITN/IRS coverage on under-five mortality rate was consistently significant in all models, except for under-five mortality IHME estimate with clustering and fixed effects (column 4). PMTCT and ART for children had significant impact on child mortality in some of the models (PMTCT in models 1, 2, 3, and 5; ART in 1, 3, 5).

## Discussion

Between 2002–08, a 66-fold increase in annual investments from $9.8 million to $651.7 million has enabled increased average ITN/IRS coverage rates from 8.3% to 33.0% to significantly impact under-five mortality rate and saving estimated number of 237,971 under-five lives in the 34 countries studied. Compared to key interventions targeted at improving child survival and health, ITNs/IRS interventions significantly reduced child mortality. Analysis of the relationship between ODA disbursement per person-at-risk and under-five mortality deaths due to malaria showed (Table 3) that high-burden countries were not consistently receiving higher ODA disbursement per person-at-risk than countries where malaria is not a major cause of child deaths.

### Input to Output

From the OECD data, it was not possible to disaggregate the amount of ODA disbursed specifically for prevention or for ITNs. Program-level costing studies, undertaken in sub-Saharan Africa suggest estimated median overall cost per LLIN distributed in 2008 to be around $7.3 (IQR: US$6.8–7.8) [26]. In sub-Saharan Africa, around 42% of Global Fund investments in malaria control up to 2008 were for prevention (32% for ITNs), 38% for treatment and 20% on health systems strengthening and program management support. Using Global Fund data, the largest external investor in malaria control, we assume that about 42% of ODA disbursement for malaria control is invested in prevention and 32% on ITNs, and the cost per net is about $7.3. Based on this assumption, we would expect 43,800 nets per $1million ODA for malaria control. This estimate falls within the 95% confidence intervals of the coefficients for our fixed effects models, in either scenario for the timing in funding disbursement and implementation (Table 4). A recent Roll Back Malaria Report [27] shows that African countries were able to rapidly invest external funds for malaria control; on average over 80% of funds invested within the year they become available. This is consistent with our results; the model which assumes that ODA disbursement is made the same year as the ITN distribution is a better fit (within R-square = 0.317 versus 0.092), indicating that much of ODA disbursement for malaria leads to ITNs distribution that same year.

### Output to Impact

Our estimate of lives saved by ITNs (0.625 lives per 1,000 additional ITNs distributed) varies from earlier estimates of 5.5 lives saved per every 1,000 children protected by ITNs [7] and 130,000 under-five lives saved [28]. The former study was a Cochrane review of cluster and individual randomized controlled trials of ITNs, and the estimate is likely to be higher than ours because the study estimated lives saved per children actually protected by ITNs while we estimated lives saved per ITNs distributed, but not necessarily used. The latter study which has used different models and methodology focused on Global Fund investments, in contrast to our analysis which explored general coverage trends and distribution not limited to the Global Fund contribution and used LiST which measures impact on the disease-attributed mortality, not on all-cause child mortality, from increased ITN/IRS coverage.

LiST measures specifically the impact of ITN and IRS coverage on reducing malaria-attributable mortality. This differs from impact on all-cause child mortality, which would result in a larger number of lives saved. Our model estimates that for every 1,000 additional ITNs distributed (i.e. not necessarily owned by household or children protected), 0.625 lives were saved in malaria-attributable mortality. By using instrumental variable, we aim to address the issue of reverse causality and more accurately measure the impact of ITN/IRS coverage on the estimated number of lives saved. Evidence suggests that in households owning at least one ITN the proportion of children under-five years who use ITNs remains low at 51% (median; range 14–68%) [8]. This may partially explain the differences observed.

### Impact of ITN/IRS coverage on Lives Saved

In countries with low-burden of child deaths due to malaria, higher average ITN/IRS coverage did not result in higher number of lives saved per population (Figure 3). The same was true for the change in ITN/IRS coverage; increase in coverage was not necessarily associated with larger number of lives saved (Figure 4). In contrast, in the countries with malaria as a major cause of child deaths, higher ITN/IRS coverage as well as coverage increase led to large numbers of lives saved per 1 million children.

These findings have major policy implications as many of the countries with malaria as a major cause of child deaths had limited increase in ITN/IRS coverage in 2002–2008 because of low investment per at-risk-person malaria (Figure 4). In spite of the relatively low investment, however, a significant number of lives is estimated to have been saved due to increases in the ITN/IRS coverage in these countries. Policies should favour higher investment per person-at-risk in countries with higher disease burden on children.

### Impact of ITN/IRS coverage on Under-five Mortality Rate

Our analysis differs from earlier studies as it combines data on ODA disbursements in malaria control (input), coverage and service delivery of malaria prevention programs (output), and their impact on under-five mortality rates along with lives saved from malaria-attributed deaths (outcome). By combining these three elements, we were able to observe the effect of investment in malaria control on child mortality to conclude that along with other key child survival interventions, increased ITNs/IRS coverage has significantly contributed to child mortality reduction since 2002.

As with earlier analyses, we used interpolated data for child mortality: a major limitation for all studies. There is an urgent need to generate micro-level data from health surveys to improve reliability of child mortality figures and robustness of results. In addition, we assume that there is a causal relationship between ITN/IRS coverage and child mortality. Statistical models demonstrate association, and not explicit causal pathways: a weakness of the approach taken.

In the final model with correction for serial correlation, we found that under-five mortality rate was significantly impacted by ITN/IRS coverage, access to quality water, immunization (DTP3 and measles), ART for children, stunting, and PMTCT, all in the expected direction. Increase in essential care during delivery and birth was negatively correlated with under-five mortality, which was the only variable that showed opposite direction from expected. This may be due to poor measures of coverage data of neonatal and maternal interventions in sub-Saharan Africa [29], [30].

By examining the cause of child deaths and LiST projection results, we showed that the lives saved estimates from LiST are strongly affected by the disease burden incorporated in the model. Lives saved estimates based on output and effectiveness data would produce results with limited insight if underlying disease burden and cause of deaths due to malaria are not appropriately incorporated in analysis. Impact varies depending on how malaria affects under-five deaths and the demography of the country, with the result that the same number of ITNs distributed in a country would have different impacts on the under-five mortality rate and lives saved from deaths attributed to malaria. We demonstrate that per person-at-risk ODA disbursements varied across countries in ways which do not reflect the malaria burden on children. Further analysis is needed to understand the reasons for the allocation patterns observed and policies produced to improve allocation of funding for malaria control. Our findings suggest the need for better prioritizing ITN/IRS scale-up in countries where malaria is a major cause of child deaths. Investments for malaria control can be allocated more efficiently by investing in countries where malaria as cause of deaths under age-five is high but per-person-at-risk malaria ODA investment remains low, saving further number of lives.

The reason for higher investment per-person-at-risk in countries with lower disease burden during 2002–2008 needs further exploration, especially for the Global Fund whose investments comprised over 70% of the ODA for malaria control for the relevant period. The Global Fund uses performance based funding throughout its investment cycle. Countries that submit high quality proposals to the Global Fund are recommended for funding for an initial period of two years, and those performing strongly against implementation plans are awarded further three years of financing. In the period 2002–2008, the sub-Saharan African countries with high-burden of child deaths due to malaria were less successful in securing funding than countries lower-burden of child deaths due to malaria. However, these countries have been able to secure substantially increased funding for 2009 and beyond following intervention by the Roll Back Malaria Partnership to assist these countries in developing strong proposals.

Effective targeting of resources is a key objective of the Global Fund, which continuously analyses its investments and refines its business model to ensure that resources reach the most affected population. This analysis, which builds on Snow et al [31], will help further refine the way in which resources are targeted. Recent revisions in the eligibility and prioritization criteria for Global Fund investments will ensure better targeting of funding to benefit countries with higher burden of malaria.

Recommendation for more efficient targeting of financial resources for malaria control against biological need and national income has been made before [31], however, our analysis takes one step further by demonstrating the association between funding allocation efficiency and number of lives saved. Additionally, we showed that ITNs play a vital role in reducing under-five mortality rate even when controlling for other child health interventions.

## Supporting Information

Box S1 Credit Reporting Systems (CRS) database of the OECD (Organisation for Economic Co-operation and Development) Development Assistance Committee (DAC).
(DOC)

Box S2 Difference between Commitments and Disbursements data.
(DOC)

Box S3 LiST: The Lives Saved Tool.
(DOC)
