# Supplementary material for: Effect of Investment in Malaria Control on Child Mortality in Sub-Saharan Africa in 2002–2008
Source: PLoS One. 2011 Jun 30;6(6):e21309. doi: 10.1371/journal.pone.0021309 (PMC3127861; doi:10.1371/journal.pone.0021309)
Supplement: Box S1 — Credit Reporting Systems (CRS) database of the OECD (Organisation for Economic Co-operation and Development) Development Assistance Committee (DAC). (DOC) [file pone.0021309.s001.doc]

**Box S1: Credit Reporting Systems (CRS) database of the OECD (Organisation for Economic Co-operation and Development) Development Assistance Committee (DAC)**

The CRS aid activity data come from donors, including the 22 member countries of the OECD’s DAC, the European Commission and other international organisations.

The bilateral donors include: Australia, Austria, Belgium, Canada, Denmark, Finland, France, Germany, Greece, Ireland, Italy, Japan, Luxembourg, Netherlands, New Zealand, Norway, Portugal, Spain, Sweden, Switzerland, United Kingdom, and United States.

Multilateral donors include: African Development Bank, Asian Development Bank, European Commission, the Global Alliance for Vaccines and Immunisation (GAVI), the Global Fund to Fight AIDS, Tuberculosis and Malaria), International Development Association (IDA), the Joint United Nations Programme on HIV/AIDS (UNAIDS), United Nations Development Programme (UNDP), United Nations Population Fund (UNFPA), and the United Nations Children’s Fund (UNICEF).
