# Supplementary material for: Effect of Investment in Malaria Control on Child Mortality in Sub-Saharan Africa in 2002–2008
Source: PLoS One. 2011 Jun 30;6(6):e21309. doi: 10.1371/journal.pone.0021309 (PMC3127861; doi:10.1371/journal.pone.0021309)
Supplement: Box S2 — Difference between Commitments and Disbursements data. (DOC) [file pone.0021309.s002.doc]

**Box S2: Difference between Commitments and Disbursements data^*^**


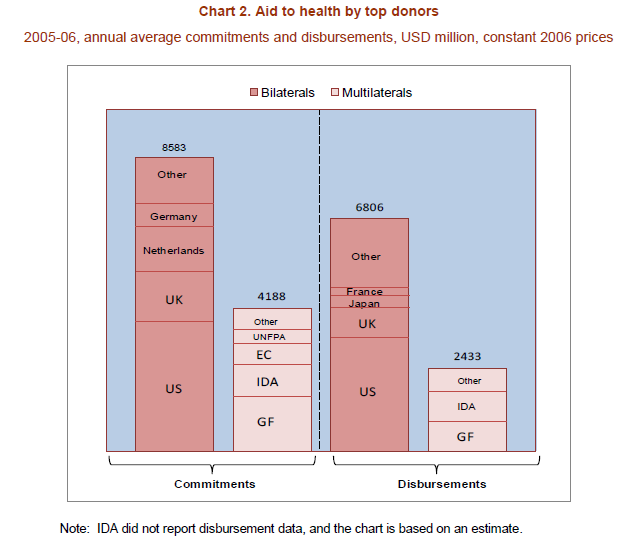


*[http://www·oecd·org/dataoecd/20/46/41453717·pdf](http://www.oecd.org/dataoecd/20/46/41453717.pdf) (Accessed 5th August 2010)
