# Supplementary material for: Effect of Investment in Malaria Control on Child Mortality in Sub-Saharan Africa in 2002–2008
Source: PLoS One. 2011 Jun 30;6(6):e21309. doi: 10.1371/journal.pone.0021309 (PMC3127861; doi:10.1371/journal.pone.0021309)
Supplement: Box S3 — LiST: The Lives Saved Tool. (DOC) [file pone.0021309.s003.doc]

**Box S3: LiST: The Lives Saved Tool**

**What is it?** LiST is a computer–based tool for estimating intervention impact· It allows users to set up and run multiple scenarios to look at the estimated impact of different intervention packages and coverage levels for their countries, states or districts· These scenarios, developed with the LiST tool, provide a structured format for program managers or ministry of health personnel to combine the best scientific information about effectiveness of interventions for maternal, neonatal and child health with information about cause of death and current coverage of interventions to inform their planning and decision–making, to help prioritize investments and evaluate existing programs· The LiST tool is meant to be used as part of the planning process—not as a replacement for planning·

**Who developed it?** A consortium of academic and international organizations, led by Institute of International Programs at the Johns Hopkins Bloomberg School, and supported by a Gates Foundation grant to the US Fund for UNICEF

**How does it work?** Running within Spectrum, an existing software package developed by the Futures Institute, the tool's LiST module works by:

- specifying the current demographic projection (either reading directly from the demographic projections of the United Nations Population Division or from national or provincial demographic projections);
- cause of death information for children under five and maternal mortality, again either standard estimates from the WHO or based on local data;
- current levels of coverage of key health interventions that affect child and maternal mortality; and
- estimated effectiveness of interventions on cause–specific neonatal, child and maternal mortality

**Why use it?**

Unique features of the tool include:

- The LiST module can be used as a stand-alone piece to investigate the impact of scaling up all of the included interventions, a subset of interventions (e.g. interventions for malaria) or a single intervention (e.g. vitamin A supplementation) and as a tool to estimate the number of lives saved by different interventions or combinations of interventions
- Information about population, current intervention coverage, and patterns/causes of mortality can be changed to run off of different national or district data if desired
- Users can easily run different scenarios and compare the effects on key variables
- Comparisons can also be made across countries creating different intervention package scenarios and target coverage levels
- Outputs and visuals can be generated in the form of line charts, bar charts, population pyramids, and tables
- A user–friendly interface will help ensure the need for minimal training to use the too

**What is the update?**

The development and recent use of LiST has been published in a special issue in the International Journal of Epidemiology[[1]](#footnote-2)

1. [http://ije·oxfordjournals·org/content/vol39/suppl_1/](http://ije.oxfordjournals.org/content/vol39/suppl_1/) (Accessed 5th August 2010)

1. [↑](#footnote-ref-2)
